# In-depth analysis of serum antibodies against Epstein-Barr virus lifecycle proteins, and EBNA1, ANO2, GlialCAM and CRYAB peptides in patients with multiple sclerosis

**DOI:** 10.3389/fimmu.2024.1487523

**Published:** 2024-12-17

**Authors:** Nicole Vasilenko, Maria P. Tieck, Tanja Michel, Sonja Schembecker, Patricia Schwarz, Anna Guenther, Christoph Ruschil, Sven Poli, Ulf Ziemann, Antje Giede-Jeppe, Gisela Gabernet, Alex Dulovic, Markus C. Kowarik

**Affiliations:** ^1^ Hertie-Institute for Clinical Brain Research, Eberhard-Karls University of Tübingen, Tübingen, Germany; ^2^ Department of Neurology & Stroke, Eberhard-Karls, University of Tübingen, Tübingen, Germany; ^3^ NMI Natural and Medical Sciences Institute at the University of Tübingen, Reutlingen, Germany; ^4^ Department of pathology, Yale School of Medicine, New Haven, CT, United States

**Keywords:** multiple sclerosis (MS), Epstein-Barr virus (EBV), EBV nuclear antigen type 1 (EBNA1), anoctamin 2 (ANO2), glial cell adhesion molecule (GlialCAM), alpha B crystallin (CRYAB)

## Abstract

**Background:**

A strong association between multiple sclerosis (MS) and Epstein-Barr virus (EBV) has been established but the exact role of EBV in MS remains controversial. Recently, molecular mimicry between EBNA1 and specific GlialCAM, CRYAB and ANO2 peptides has been suggested as a possible pathophysiological mechanism. The aim of this study was to analyse anti-EBV antibodies in MS patients against (I) EBV lifecycle proteins, (II) putative cross-reactive peptides, and (III) during treatment.

**Methods:**

In this retrospective cross-sectional study, 258 serum samples were included consisting of EBV-negative (n = 25) and EBV-positive (n = 36) controls, 192 MS samples including untreated relapsing-remitting MS (RRMS) with and without relapses, secondary progressive MS (SPMS) and primary progressive MS (PPMS) patients, and 106 patients on 8 different treatment regimens. IgG and IgM antibody titers against EBV docking/fusion proteins (gp350, gh/gp42, gh/gL/gp42), immediate early antigen (BZLF1), early antigens (EA p85, EA P138, EA P54), capsid antigens (VCA P18, VCA P23, VCA gp125) and late antigens (EBNA1) were measured. Specific EBNA1 and GlialCAM, CRYAB and ANO2 peptides were synthesized and also incorporated in our custom magnetic bead based multiplex assay.

**Results:**

We observed significantly elevated IgG antibody titers in EBV-positive controls, RRMS with and without relapse, SPMS and PPMS patients for all lifecycle antigens except for several early antigens when compared to EBV-negative controls. Significantly higher IgG antibody titers were observed in RRMS patients for fusion proteins and EBNA1 peptides when compared to EBV-positive controls. An MS specific response was observed for ANO2 but not for GlialCAM or CRYAB. No significant treatment effects or a specific IgM response were detectable.

**Conclusion:**

The MS-specific, differential antibody response to EBV antigens confirms an altered immunological response to EBV in MS patients. EBV reactivation does not appear to play an important role in MS pathogenesis and no differential antibody signatures were observed between MS disease phases. The MS-specific anti-ANO2 antibody response suggests a potential role for EBNA1 as an antigenic driver, although the exact role of anti-ANO2 antibodies needs to be determined. The precise pathophysiological role of EBV in MS remains uncertain and requires further investigation.

## 1 Introduction

Epstein-Barr virus (EBV), also known as human herpesvirus 4, is a ubiquitous virus that infects approximately 90% of adults, as evidenced by the presence of EBV-specific antibodies (1). While EBV is recognized as an oncogenic virus involved in the pathogenesis of B-cell lymphoma, nasopharyngeal carcinoma, and some gastric cancers (2, 3), strong associations with autoimmune disorders such as systemic lupus erythematosus (SLE), rheumatoid arthritis (RA) and multiple sclerosis (MS) have also been identified (2). Similarly to other herpesviruses, EBV has a productive lytic cycle and a latent phase. The lytic infection of EBV usually occurs in epithelial cells, resulting in the generation of progeny virions for further transmission, whereas latent infection is primarily established in B cells (2, 3). Serological testing to identify EBV serostatus assess antibodies against EBV nuclear antigen type 1 (EBNA1), viral capsid antigen (VCA), and early antigen (EA). Following primary infection, antibodies to VCA and EBNA1 persist for lifetime (4, 5), whereas EA and BZLF1 antibodies are expressed during lytic cycles. Consequently, high anti-VCA, anti-EA, and anti-BZLF1 Ig titers are regarded as indicators of EBV reactivation (4). Antibodies against gp350, only appear during convalescent phase showing neutralizing functions (6), with both anti-gp350/220 and anti-gh/gp42 complex antibodies suggested as exerting neutralizing effects (7).

Numerous serological studies into MS suggest that a primary EBV infection is a strong predisposing factor (8, 9) with increased EBNA1 antibodies being detectable several years before the clinical onset of MS (10–12). Follow-up studies post-MS diagnosis could not identify clear associations between either EBNA1 or VCA antibody titers and disease progression, clinical or radiological activity (13, 14). Another study reported a significantly elevated percentage of MS patients (48%) with increased EA IgG antibodies compared with matched healthy controls, while only 4% were VCA IgM positive. Thus, active viral replication has been postulated in a subset of MS patients, however this viral activation was unrelated to disease activity (15). Among other EBV antibodies in MS patients, BZLF1 antibodies, while slightly elevated compared to matched controls, were not found to be predictive for MS (16), whereas gp350 antibodies are elevated in MS patients (17, 18). To our knowledge, no studies have been reported on gh/gp42 complex antibodies in MS.

Several MS treatments have been shown to exert differential effects on B cells with a consistent reduction of peripheral memory B cells which serve as a reservoir for EBV (19–21). One might speculate that a reduction of EBV load could in return decrease EBV antibodies and T cell responses (22, 23), as evidenced by decreases in EBNA1 antibodies following treatment with ocrelizumab and teriflunomide but not with interferon beta (23–27). Similarly, natalizumab has been shown to decrease gp350 IgG titers (18).

Despite the consistent establishment of a strong association between EBV and MS, the exact role of EBV in multiple sclerosis remains controversial. Different hypotheses have been discussed during the last years including EBV reactivation as an immunological trigger, EBV as a direct antigenic driver of MS, or an alteration of immunological reactions through EBV supporting CNS-directed autoimmune responses (28, 29). In the context of potential pathomechanisms in MS, EBNA1 has been the subject of the most comprehensive investigation to date. Peptidome studies, including those examining peptides derived from a wide range of EBV proteins, have revealed strikingly elevated IgG antibody levels against a variety of latent EBV proteins, particularly against EBNA1, in individuals diagnosed with MS as well as in those exhibiting early signs of MS (30). Furthermore, elevated antibody titers against EBNA1 peptides in the glycine-alanine repeat region and the C-terminal domain have been identified as potential target regions, given their increased prevalence in MS patients (31–33). Recent studies have reported cross-reactivity (molecular mimicry) of antibodies to EBNA1 that are also directed against anoctamin 2 (ANO2) (34), alpha B crystallin (CRYAB) (35), or glial cell adhesion molecule (GlialCAM) (36). Besides the detection of antibodies against GlialCAM and EBNA1 in a subset of MS patients, injection of another EBNA1 peptide into mice with experimental autoimmune encephalomyelitis (EAE) exacerbated CNS autoimmunity (36). Another study also detected GlialCAM antibodies but no significant differences were observed between EBNA1^high^ controls and MS patients (37). As another putative MS antigen, CRYAB has been shown to be one of the most abundant gene transcripts in early active MS lesions (38). Furthermore, administration of recombinant CRYAB peptides ameliorated EAE models (38) and CRYAB has been shown to serve as a potential antigen for serum and CSF antibodies in MS patients (35, 38, 39). More recently, a cross-reactivity between CRYAB and EBNA1 antibodies has been postulated (35). An increased antibody reactivity against the chloride channel protein ANO2 could also be identified in MS patients compared with controls (40). ANO2 expression was found near and inside of MS lesions suggesting a potential role in MS etiopathogenesis (40). More recently the same group was able to establish molecular mimicry between ANO2 (AA 140-149) and EBNA1 (AA 431 – 440) and confirmed an increased MS risk for ANO2-seropositive individuals, HLA-DRB1*15:01 carriage, and high anti-EBNA1 antibody levels (34).

Previous antibody screening studies against EBV in MS have primarily focused on EBNA1 or the EBNA complex, with only a few other EBV proteins investigated at the same time. A variety of EBV lifecycle proteins have been analysed in patients prior to the onset of MS, but not after a MS diagnosis, across different MS types or during acute relapse. Furthermore, these data predominantly comprise IgG antibody measurements, with only limited data pertaining to IgM antibody measurements (15, 41–44). In our cross-sectional retrospective study, we aimed to provide an in-depth analysis of EBV IgG and IgM antibodies against different EBV lifecycle proteins, and of treatment effects on EBV antibody titers to obtain a complete picture regarding possible EBV reactivation and the specific EBV immune response in MS patients. Furthermore, we were interested whether we could confirm the presence of cross-reactive antibodies against defined EBNA1 peptides and their homologous GlialCAM, ANO2 and CRYAB peptide counterparts. In consequence, we present a comprehensive analysis of multiple EBV-associated antigens that has not been previously undertaken to such an extent.

## 2 Methods

### 2.1 Standard protocol approvals and consent forms

This study was approved by the ethics committee at the Medical Faculty of the Eberhard Karls University and at the University Hospital of Tübingen in accordance with the Declaration of Helsinki (Ethic approval number 204/2018B02). Written informed consent was obtained from all patients included in the study.

### 2.2 Study design and patient population

We performed a retrospective cross-sectional analysis of MS patients’ serum samples who visited our clinic between 2018 to 2024. The MS diagnosis was established according to the 2017 and 2010 McDonald criteria (45, 46). Serum samples were obtained during a routine clinical blood draw according to our in-house routine and stored at -80°C. The following MS patients groups were selected for further analyses: untreated RRMS patients during stable disease (n = 45), untreated RRMS patients during relapse (n = 39, patients received treatment with methylprednisolone), untreated SPMS patients (n = 17), untreated PPMS patients (n = 10), RRMS patients receiving treatment with glatiramer acetate (GLAT, n = 11), teriflunomide (TER, n = 13), dimethyl fumarate (DMF, n = 23), cladribine (CLAD, n = 19), ozanimod (OZA, n = 14), natalizumab (NAT, n = 10) or ocrelizumab (OCR, n = 15). Relapses were defined as subacute new or worsening clinical symptoms that last for at least 24 h and were separated from a previous attack by a minimum of 30 days. Serum samples in this cohort were collected if the patient met these criteria on admission. Stable disease was defined as no disease activity in the last 90 days. The untreated RRMS patient cohort consisted of 29 patients who had never been treated with immunomodulatory/-suppressive medications, while the remaining 16 patients had been untreated for at least 90 days or had discontinued treatments in accordance with the recommended washout periods for the specific medication. The majority of patients who received treatment had previously undergone therapy with either interferons or glatiramer acetate as the most recent treatment regimen (11/16). Healthy EBV-negative (n = 23) and EBV-positive controls (n = 24) were obtained from Central BioHub. Additional healthy EBV-positive controls (n = 12) and healthy EBV-negative controls (n = 2) were provided by our laboratory. EBV-positive and -negative controls were defined by commercially available ELISA-Test performed by the NMI. Clinical data including age and gender were obtained for all patients. MS patients were further characterized on disease duration, EDSS score, treatment duration and number of previous treatments. An overview of patient characteristics is given in **Table 1** and additional data is shown in **Supplementary Table 1**.

**Table 1:** Description of characteristics within different patient groups in this study.

	Patient numbers	Age Median in years (min – max)	Gender (f / m)	Disease duration Median in months (min – max)	Treatment duration Median in months (min – max)	Number of previous DMTs Median (min – max)	Patients with relapse (n)	EDSS score Median (min – max)
EBV-negative control	25	14 (1 – 32)	10 / 15 (40% / 60%)	–	–	–	–	–
EBV-positive control	36	25 (6 – 75)	21 / 15 (42% / 58%)	–	–	–	–	–
Untreated RRMS during relapse	39	26 (19 – 49)	30 / 9 (77% / 23%)	1 (0 – 65)	–	0 (0 – 0)	39	1.5 (0 – 4.5)
Untreated RRMS	45	30 (20 – 62)	34 / 11 (76% / 24%)	6.5 (0 – 366)	–	0 (0 – 5)	0	2 (0 – 6.5)
Untreated SPMS	17	50 (32 – 71)	10 / 7 (59% / 41%)	158 (16 – 405)	–	1 (0 – 4)	0	4.5 (3 – 7)
Untreated PPMS	10	52.5 (40 – 62)	5 / 5 (50% / 50%)	26 (7 – 166)	–	0 (0 – 1)	0	4 (1.5 – 6.5)
RRMS glatiramer acetate	11	26 (20 – 47)	10 / 1 (91% / 9%)	12 (1 – 62)	7 (2 – 25)	0 (0 – 2)	6	1.5 (0 – 3.5)
RRMS teriflunomide	13	46 (21 – 59)	3 / 10 (23% / 77%)	16 (5 – 148)	6 (3 – 36)	0 (0 – 1)	5	2 (0 – 2.5)
RRMS dimethyl fumarate	23	36 (21 – 52)	16 / 7 (70% / 30%)	16.5 (7 – 89)	11 (6 – 56)	0 (0 – 2)	7	1.5 (0 – 5.5)
RRMS cladribine	19	30 (22 – 59)	14 / 5 (74% / 26%)	64 (24 – 318)	17 (6 – 49)	1 (0 – 5)	3	2.5 (1.5 – 7)
RRMS ozanimod	14	31 (20 – 44)	8 / 6 (57% / 43%)	10 (6 – 24)	6 (6 – 23)	0 (0 – 0)	6	1.5 (0 – 2)
RRMS natalizumab	10	31 (19 – 52)	9 / 1 (90% / 10%)	146 (25 – 343)	33 (5 – 113)	2 (1 – 4)	3	2 (0 – 6.5)
RRMS ocrelizumab	15	32 (21 – 62)	10 / 5 (67% / 33%)	56 (5 – 319)	12 (2 – 48)	1 (0 – 4)	7	2.5 (1 – 3.5)

EBV, Epstein-Barr virus; RRMS, relapsing remitting multiple sclerosis patients; SPMS, secondary progressive multiple sclerosis; PPMS, primary progressive multiple sclerosis; EDSS, Expanded Disability Status Scale.

### 2.3 EBV multiplex assay

For the in-house multiplex assay, we included EBV proteins that represent the different phases of EBV lifecycle including docking (gp350/220), fusion (gh/gL/gp42 and gH/gp42), immediate early antigen (BZLF1), early antigens (EA P54, EA P138, EA P85), viral capsid antigens (gp125, P23, P18) and EBV nuclear antigens (EBNA1). Furthermore, we synthesized specific EBNA1 peptides (AA386-405, AA393-412, AA425-444) that have previously been shown to potentially provide cross reactive binding sites for autoantibodies against specific GlialCAM (AA370-389), CRYAB (AA2-21) and ANO2 (AA134-153) peptides. The GlialCAM peptide was phosphorylated as described by Lanz and colleagues (36), for further details on specific peptides please refer to **Supplementary Table 2**.

EBV proteins were sourced commercially from various providers (**Supplementary Table 2** for details) and coupled to spectrally distinct populations of MagPlex Microspheres (Luminex Corporation) by EDC-sNHS chemistry. Coupling was performed at room temperature using a KingFisher96 (ThermoFisher Scientific). In brief, uncoupled beads were vortexed thoroughly and sonicated for 3 minutes (mins). 500 µL of each individual bead ID was then added to individual wells of a 96 deep well plate with 42 µL of 0.065% Triton X-100 in ddH_2_O. Beads were then washed twice with 250 µL of activation buffer (100 mM Na_2_HPO_4_ (pH 6.2) + 0.005% Triton X-100), then activated for 20 mins in 150 µL of 5 mg/mL EDC and 5 mg/mL s-NHS in activation buffer. Beads were then washed again using 250 µL of coupling buffer (50 mM MES (pH 5.0) + 0.005% Triton X-100) and then incubated for 2 h in 125 µL of antigen-containing coupling buffer. The exact concentration of each antigen used is provided in **Supplementary Table 2** and was determined in pilot testing. Following this, beads were washed twice with 250 µL wash buffer (PBS + 0.005% Triton X-100) and then resuspended in 1 mL storage buffer (CBS + 0.05% ProClin) and stored at 4°C until required.

Peptides were ordered from a commercial source (Intavis) with a biotin-TtdS modification to enable coupling to beads. In brief, streptavidin was coupled to individual bead populations using EDC-sNHS chemistry as above. 200 µL of individual bead populations was then added to individual wells of a 96 deep well plate and washed with 200 µL of CBST (0.1% Tween-20 in CBS). The peptides were then diluted to 1 mM in 250 µL of CBST, added to each well and incubated for 2 h. Following 2 washes with 300 µL of CBST, 250 µL of 2.5 µM deactivated biotin-sNHS was added to each well and incubated for 1 h. Beads were then washed twice more with 300 µL CBST and then stored in 200 µL of storage buffer at 4°C until required. Prior to measurement, all coupled beads were combined together in a 25x bead mix (20,000 beads per ID/mL).

For measurement, samples were diluted 1:200 in assay buffer (47) inside a sterile workbench. 25 µL of diluted sample was then added to individual wells of a 96 half-well plate (Corning) with 25 µL of 2x bead mix and incubated for 2 h at 20°C, 750 rpm in a light protected thermomixer. Following this, unbound antibodies were removed by washing 3x with wash buffer (1x PBS, 0.05% Tween20) using an automated microplate washer (Biotek 405TS). To enable measurement of both IgG and IgM, beads were then resuspended in 100 µL of wash buffer and then split in two, with 50 µL transferred to a fresh half-well plate. Bound antibodies were then detected using either 3 µg/mL RPE-huIgG (Dianova) or 5 µg/mL RPE-huIgM (Dianova) by incubation for 45 mins at 20°C, 750 rpm on a light-protected thermomixer. Plates were then washed 3x again, with beads resuspended in 100 µL of wash buffer and resuspended by shaking at 20°C, 1000 rpm for 3 mins. Plates were then measured using a FLEXMAP3D (Luminex Corporation) using the following settings: 80 µL (timeout 60 sec), 100 events, Gate 7500-15000 and Report Gain: Standard PMT. As controls, blanks (assay buffer) and confirmed EBV-positive and EBV-negative samples were included on each plate. In-well QC beads for IgG/IgM and sample addition were also included and with mean fluorescence intensity (MFI) values required to reach pre-determined thresholds for positivity for measurement to be considered valid. Per antigen, samples were classified as positive for IgM or IgG when they exceeded the mean MFI of the negative cohort plus 6x the standard deviation. To demonstrate assay performance, clinical validation data (comparison of commercial ELISAs and multiplex assay for EA IgG, VCA IgG and IgM and EBNA-1 IgG) as well as basic technical validation (inter-assay variation) are included as **Supplementary Figure 1**. Samples used in this validation were obtained from commercial biobanks (Central BioHub and InventDiagnostika).

### 2.4 Statistics

For statistical analyses and visualization GraphPad Prism (Version10.1.1) was applied. Since the data did not consistently show a normal distribution with the Kolmogorov-Smirnov test, the non-parametric tests for data analysis were used. In order to test for significances between minimum three groups the Kruskal Wallis test was performed using Dunn’s correction for multiple testing. In order to test for significances between two groups the Mann-Whitney U test was performed using Šídák correction for multiple independent testing. In addition, the Spearman correlation with Bonferroni correction for further data analyses was applied.

## 3 Results

### 3.1 MS patients, control groups and demographic data

Demographic differences between patients (treated and untreated) and control groups are provided as **Table 1** and in addition **Supplementary Table 1**. All groups, with the exception of the EBV-negative controls (40%), untreated PPMS (50%) and RRMS teriflunomide (23%) patients were predominantly female (**Table 1**). The imbalanced distribution on sex had no effect on the Ig measurements, as no significant differences were found between males and females against the various antigens (data not shown). No major differences between the groups were observed regarding ethnicity. Due to the early age at which the majority of individuals are infected with EBV, the EBV-negative controls were significantly younger than all other groups (**Supplementary Figure 2**). No significant differences were seen between treatment groups (**Supplementary Figure 3**). Untreated SPMS and PPMS patients were significantly older than untreated RRMS patients (**Supplementary Figure 2**). Untreated MS patients during relapse had a significantly shorter disease duration when compared to untreated RRMS, SPMS, and PPMS, with untreated SPMS also having a significantly increased disease duration when compared to untreated RRMS patients (**Supplementary Figure 2**). Among treated groups, CLAD, NAT and OCR had the longest disease durations (**Supplementary Figure 3**). Untreated patients with SPMS and PPMS had significantly higher EDSS values compared to RRMS groups (**Supplementary Figure 2**), whereas among treated groups, OZA had significantly lower EDSS compared to CLAD, OCR and untreated RRMS (**Supplementary Figure 3**). Untreated SPMS patients had a significantly higher number of DMTs than the other untreated patient groups, and untreated RRMS patients a significantly higher number of DMTs than RRMS during relapse (**Supplementary Figure 2**). CLAD and NAT treated MS patients had significantly more DMTs than GLAT, TER, DMF, OZA and untreated MS patients (**Supplementary Figure 3**). In line with this, treatment duration was significantly longer in NAT treated patients compared to those receiving GLAT, TER or OZA (**Supplementary Figure 3**). Among all treated groups, a subset of patients experienced a relapse during the time of blood sampling.

### 3.2 IgG antibody response against EBV lifecycle peptides

**Figure 1:**
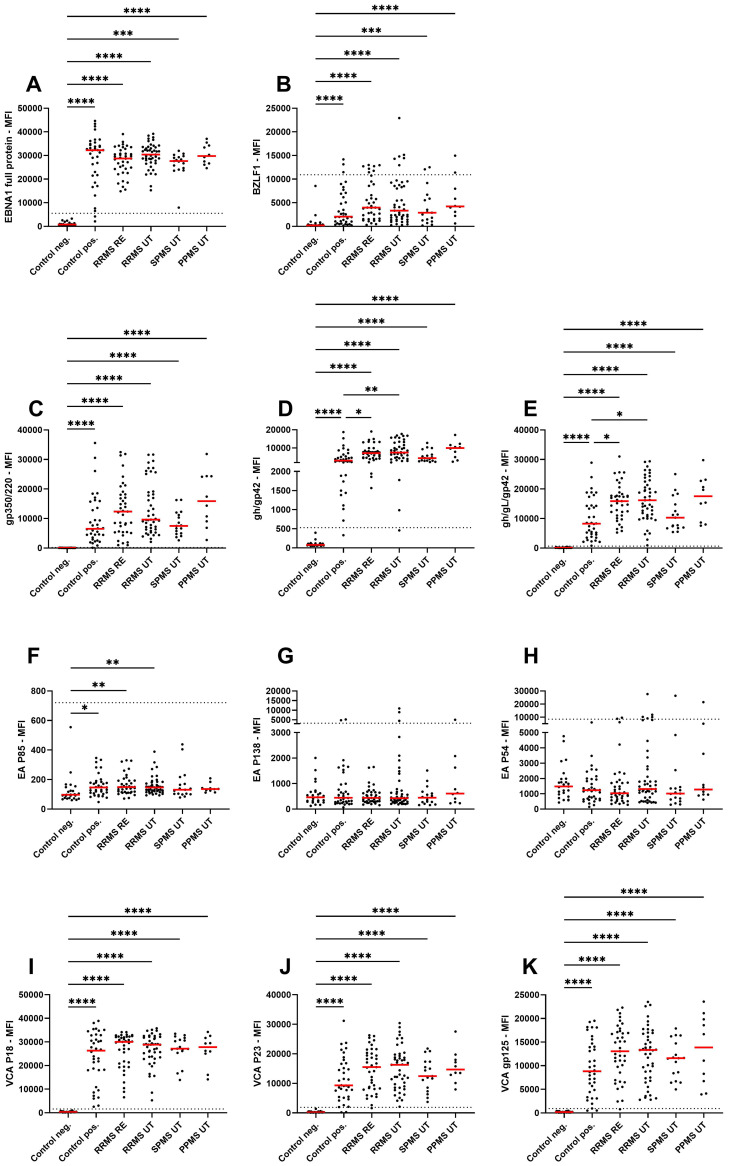
IgG antibody titers against EBV lifecycle peptides. Mean fluorescence intensities (MFI) of the serum antibody response against **(A)** EBNA1 late antigen, **(B)** BZLF1 immediate early antigen, **(C)** gp350/220 docking protein, **(D)** gH/gp42 and **(E)** gh/gL/gp42 fusion proteins, **(F)** EA P85, **(G)** EA P138, **(H)** EA P54 early antigens, **(I)** VCA P18, **(J)** VCA P23, and **(K)** VCA gp125 viral capsid antigens are shown for EBV-negative controls, EBV-positive controls, relapsing remitting multiple sclerosis patients during relapse (RRMS RE), untreated and without relapse (RRMS UT), untreated secondary progressive multiple sclerosis (SPMS UT) and untreated primary progressive multiple sclerosis (PPMS) are shown. Kruskal-Wallis test with Dunn’s correction was applied for statistical testing (* p < 0.05, ** p < 0.01, *** p < 0.001, **** p < 0.0001). The horizontal dotted line indicates the cut-off for IgG seropositivity.

Antibody titers towards various EBV lifecycle proteins were analysed within the study cohort to assess potential differences between different MS disease phases and phenotypes (**Figure 1**). For all antigens, except early antigens, EBV-positive controls, untreated RRMS with and without relapse, SPMS and PPMS patients had significantly higher titers compared to EBV-negative controls (**Figure 1**). Within the early antigens, significantly higher antibody titers for EA P85 were observed for EBV-positive controls, RRMS patients with and without relapse when compared to EBV-negative controls whereas no significant differences were observed for EA P138 and EA P54. Within EBV-positive controls and MS patient groups, antibody titers for the fusion protein complexes (gh/gL/gp42 and gH/gp42) were significantly higher in untreated RRMS patients with and without relapse when compared to EBV- positive controls (**Figures 1D, E**). No significant effects were observed between the different untreated MS subgroups. Next, to assess the effect of treatment on EBV antibodies, we assessed Ig titers in response to treatment with GLAT, TER, DMF, CLAD, OZA, NAT and OCR, finding no significant differences when compared between each other and to untreated RRMS (**Supplementary Figure 4**). Among potential co-factors such as age, disease duration, EDSS, number of previous treatments and treatment duration, only age significantly correlated with gp350/220, VCA P18 and VCA P23 (positive correlation) (**Supplementary Figure 5**). Between the different lifecycle proteins, positive correlations were identified both within and between docking, fusion and viral capsid antigen antibodies (**Supplementary Figure 5**).

### 3.3 IgG antibody response against EBNA1 peptides and GlialCAM, CRYAB and ANO2 peptides

**Figure 2:**
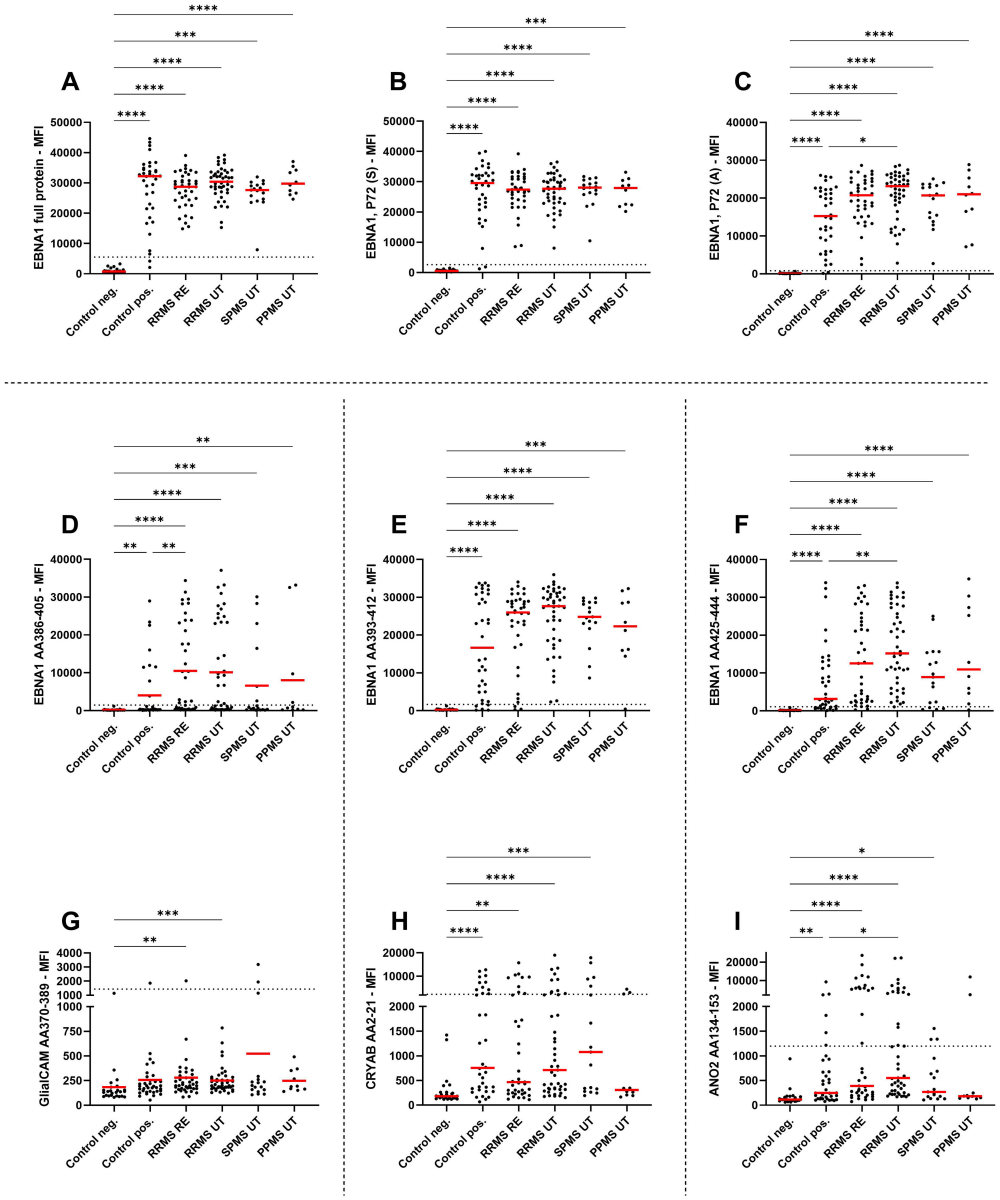
IgG antibody titers against EBNA peptides and potential cross reactivity against related peptides. Mean fluorescence intensities (MFI) of the serum antibody response against **(A)** EBNA1 full antigen, **(B)** EBNA1 (Serion), **(C)** EBNA1 (Aviva), **(D)** EBNA1 AA386-405, **(E)** EBNA1 AA393-412, **(F)** EBNA1 AA425-444, **(G)** GlialCAM AA370-389, **(H)** CRYAB AA2-21, and **(I)** ANO2 AA134-153 are shown for EBV-negative controls, EBV-positive controls, relapsing remitting multiple sclerosis patients during relapse (RRMS RE), untreated and without relapse (RRMS UT), untreated secondary progressive multiple sclerosis (SPMS UT) and untreated primary progressive multiple sclerosis (PPMS) are shown. Kruskal-Wallis test with Dunn’s correction was applied for statistical testing (* p < 0.05, ** p < 0.01, *** p < 0.001, **** p < 0.0001). The horizontal dotted line indicates the cut-off for IgG seropositivity.

Given the potential cross-reactivity of anti-EBV antibodies, EBNA1 peptides (AA386-405, AA393-412, AA425-444), GlialCAM (AA370-389), CRYAB (AA2-21), and ANO2 (AA134-153) peptides were also included in the multiplex assay (**Figure 2**). Due to the generally low signal observed against the peptides GlialCAM, CRYAB and ANO2, normal values were determined and subsequently the percentage of MS patients with positive antibody titers were assessed (**Table 2**).

**Table 2:** IgG positivity response rate against EBNA1 proteins, peptides and related peptides.

	EBNA1 AA386-405	GlialCAM AA370-389	EBNA1 AA393-412	CRYAB AA2-21	EBNA1 AA425-444	ANO2 AA134-153	EBNA1 full protein	EBNA1, p72 (S)	EBNA1, p72 (A)
EBV-negative control (n = 25)	0 / 0%	0 / 0%	0 / 0%	0 / 0%	0 / 0%	0 / 0%	0 / 0%	0 / 0%	0 / 0%
EBV-positive control (n = 36)	9 / 25%	1 / 2.8%	31 / 86.1%	11 / 30.6%	26 / 72.2%	6 / 16.7%	34 / 94.4%	34 / 94.4%	34 / 94.4%
RRMS UT RE (n = 39)	22 / 56.4%	1 / 2.6%	36 / 92.3%	9 / 23.1%	37 / 94.9%	15 / 38.5%	39 / 100%	39 / 100%	39 / 100%
RRMS UT (n = 45)	23 / 51.1%	0 / 0%	45 / 100%	10 / 22.2%	44 / 97.8%	17 / 37.8%	45 / 100%	45 / 100%	45 / 100%
SPMS UT (n = 17)	6 / 35.3%	2 / 11.8%	17 / 100%	5 / 29.4%	13 / 76.5%	3 / 17.6%	17 / 100%	17 / 100%	17 / 100%
PPMS UT (n = 10)	4 / 40.4%	0 / 0%	9 / 90%	3 / 30%	9 / 90%	2 / 20%	10 / 100%	10 / 100%	10 / 100%
RRMS GLAT (n = 11)	7 / 63.6%	0 / 0%	11 / 100%	4 / 36.4%	10 / 90.9%	7 / 63.6%	11 / 100%	11 / 100%	11 / 100%
RRMS TER (n = 13)	5 / 38.5%	0 / 0%	9 / 69.2%	2 / 15.4%	12 / 92.3%	3 / 23.1%	13 / 100%	13 / 100%	13 / 100%
RRMS DMF (n = 23)	8 / 24.8%	2 / 8.7%	20 / 87.0%	9 / 39.1%	19 / 82.6%	7 / 30.4%	23 / 100%	23 / 100%	23 / 100%
RRMS CLAD (n = 19)	7 / 36.8%	0 / 0%	17 / 89.5%	5 / 26.3%	15 / 78.9%	6 / 31.6%	19 / 94.7%	19 / 94.7%	19 / 94.7%
RRMS OZA (n = 14)	6 / 42.9%	1 / 7.1%	14 / 100%	1 / 7.1%	11 / 78.6%	4 / 28.6%	14 / 100%	14 / 100%	14 / 100%
RRMS NAT (n = 10)	4 / 40%	0 / 0%	9 / 90.0%	4 / 40%	8 / 80%	2 / 20%	10 / 100%	10 / 100%	10 / 100%
RRMS OCR (n = 15)	9 / 60%	0 / 0%	14 / 93.3%	4 / 26.7%	13 / 86.7%	4 / 26.7%	15 / 100%	15 / 100%	15 / 100%

Samples were classified as positive when the MFI was greater than the mean of the negative control group plus 6x SD. Data is provided as both number who are positive and the corresponding percentage. EBV, Epstein-Barr virus; UT, untreated; RRMS, relapsing remitting multiple sclerosis patients; GLAT, glatiramer acetate; TER, teriflunomide; DMF, dimethyl fumarate; CLAD, cladribine; OZA, ozanimod; NAT, natalizumab; OCR, ocrelizumab; S, Serion; A, Aviva.

All MS patients were tested positive for EBNA1 antibodies (**Table 2**), with EBNA1 titers being significantly higher in EBV-positive controls and all untreated MS patient groups compared to EBV-negative controls (**Figure 2A**). To confirm this, we assessed EBNA1 titers against two further commercially available EBNA1 p72 proteins, resulting in almost identical results (**Figures 2B, C**). For one of the EBNA1 p72 proteins (P72, A), a further significant difference was identified between RRMS relapse patients and the EBV-positive control group (**Figure 2C**). Significantly elevated antibody titers were also observed against all specific EBNA1 peptides (EBNA1 AA386-405, EBNA1 AA393-412, EBNA1 AA425-444) in EBV-positive controls and all untreated MS patients when compared to EBV-negative controls (**Figures 2D–F**). However, the percentage of positive patients was generally lower when compared to the aforementioned commercially available EBNA1 protein antigens and ranged from 25%-86% in EBV-positive controls and 35%-100% in the different MS patient groups (**Table 2**).

EBNA1 AA386-405 antibodies were also significantly higher in RRMS patients during relapse when compared to EBV-positive controls (**Figure 2D**). For the corresponding GlialCAM peptide, titers were minimal with significantly increased values only found for untreated RRMS patients with and without relapse when compared to EBV-negative controls (**Figure 2G**). Overall positivity of GlialCAM was low among both, MS patients (0%-11.8%) and EBV-positive controls (2.8%, **Table 2**).

**Figure 3:**
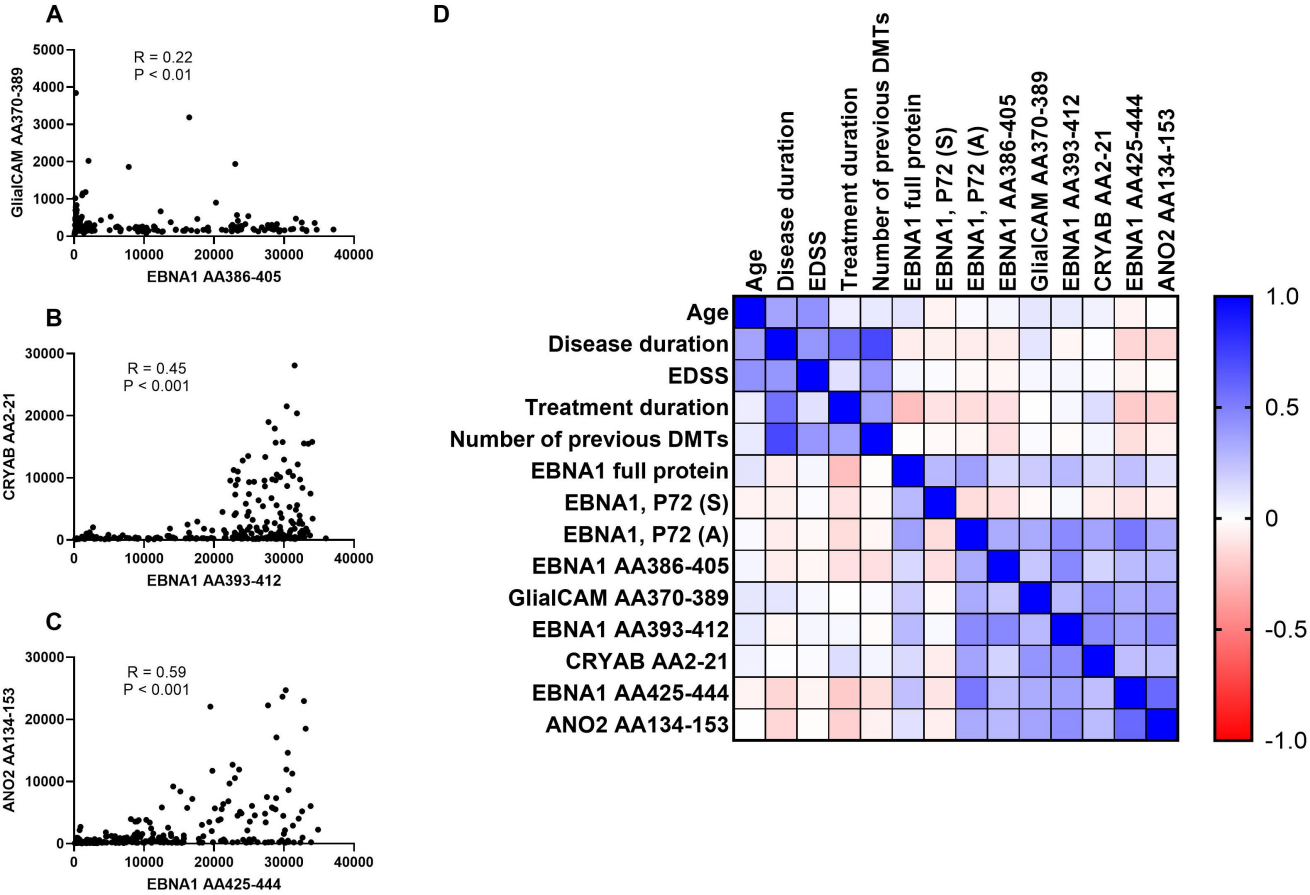
Correlations between EBNA peptides and potentially cross-reactive peptides and correlation matrix. Correlations between **(A)** EBNA1 AA386-405 and GlialCAM AA370-389, **(B)** EBNA1 AA393-412 and CRYAB AA2-21, **(C)** EBNA1 AA425-444 and ANO2, and **(D)** the correlation matrix between all EBNA1 and related peptides as well as additional patient characteristics are shown. The Spearman correlation test was employed for the purpose of statistical analysis. DMTs, disease-modifying treatments; EDSS, Expanded Disability Status Scale.

Titers against CRYAB were significantly higher for EBV-positive controls and all untreated MS patient groups except for PPMS when compared to EBV-negative controls (**Figure 2H**). No significant differences were detectable between RRMS patients and EBV-positive controls (**Figure 2H**). The percentage of CRYAB positive patients was similar between EBV-positive controls (30.6%) and untreated MS patients (22.2%-30%, **Table 2**). A similar pattern of increased titers for EBV-positive controls and all untreated MS patient groups except for PPMS when compared to EBV-negative controls was found for ANO2, although titers for RRMS without relapse were also significantly higher than the EBV-positive controls (**Figure 2I**). Positive antibody titers were also observed more frequently among untreated MS patient groups (17.6%-38.5%), especially in untreated MS patients with (38.5%) and without relapse (37.8%), compared to EBV-positive controls (16.7%, **Table 2**). No significant differences were seen between treatment groups for either EBNA1 proteins or peptides or GlialCAM, CRYAB and ANO2 peptides (**Supplementary Figure 6**). No significant correlations were seen between EBNA1 and related peptides for any co-factors (**Figure 3D**). Overall, significant positive correlations were seen between specific EBNA1 peptides and GlialCAM, CRYAB and ANO2 (**Figure 3**).

### 3.4 IgM antibody response against EBV lifecycle proteins, EBNA1 peptides and GlialCAM, CRYAB and ANO2 peptides

Lastly, to assess evidence of possible reactivation, we analysed the presence of IgM antibodies against the same EBV lifecycle proteins within the study cohort. Samples were classified as IgM positive based upon defined COs (see methods for details). In general, a low IgM positivity frequency of ≤ 10% was observed for most EBV proteins/peptides in the different patient cohorts, with only untreated PPMS patients having > 10% positivity for gp350/220 (20%), gh/gL/gp42 (30%) and VCA gp125 (30%, **Supplementary Table 3**). Among treatments, only anti-BZLF1 in OZA treated patients (21.4%) had an IgM positivity rate > 10% (**Supplementary Table 3**). No differences in IgM positivity were seen for the EBNA1, GlialCAM, CRYAB or ANO2 peptides between different groups or treatments, with only small differences seen between the different commercial EBNA1 proteins (**Supplementary Table 4**).

## 4 Discussion

Multiple sclerosis has been strongly associated with an elevated EBV serological response to EBNA1 years before clinical onset. With regard to the pathophysiological role of EBV infection, immune responses against EBV may induce an autoimmune reaction by molecular mimicry or alternatively, alter immunological responses by e.g. establishing a lifelong latent infection in B cells. The objective of this study was to investigate whether antibodies indicative of different stages of the virus lifecycle show distinct titers during different MS disease phases including relapses. Furthermore, we examined the occurrence of antibodies against EBNA1 peptides and putative cross-reacting peptides (GlialCAM, CRYAB and ANO2) that might be involved in molecular mimicry. Our study provides an in-depth analysis of the antibody response against a multitude of EBV antigens in MS, thus facilitating a more complete picture of the humoral immune responses associated with EBV.

In line with other studies, all of the MS patients in our cohort had detectable antibodies against EBNA1 (2, 48). Additionally, a consistent antibody response was found against VCA antigens which has also been described in previous studies (13, 14). MS patients also showed antibody titers against the docking protein gp350/220 and the fusion proteins gh/gp42 and gh/gL/gp42 with significantly elevated antibody titers against the latter two antigens when compared to EBV-positive controls. No antibody titers were found against the early antigens P138 and P54 while antibodies against EA P85 were found in RRMS patients and EBV-positive controls which contrasts with previous results showing an elevated percentage of MS patients with elevated antibody titers against early antigens (p54) when compared to matched controls (15, 49). BZLF1 (immediate early antigen) antibody titers were also found in EBV-positive controls and all MS patient groups, but did not show significant differences between these two groups which is in accordance with the data by Ruprecht and colleagues (31). As expected, no consistently elevated IgM antibodies were found for the different antigens and within the different groups (15, 49). In our cross-sectional design, no significant treatment effects of GLAT, TER, DMF, CLAD, OZA, NAT or OCR were observed on antibody titers. These results contrast with longitudinal studies that show a reduction of anti EBNA1 antibodies following OCR and TER treatments (23–27). Given the considerable inter-individual variation in serum antibody titers against defined targets, it is possible that distinct longitudinal changes in individual MS patients may not be reflected in our cross-sectional analysis.

**Figure 4:**
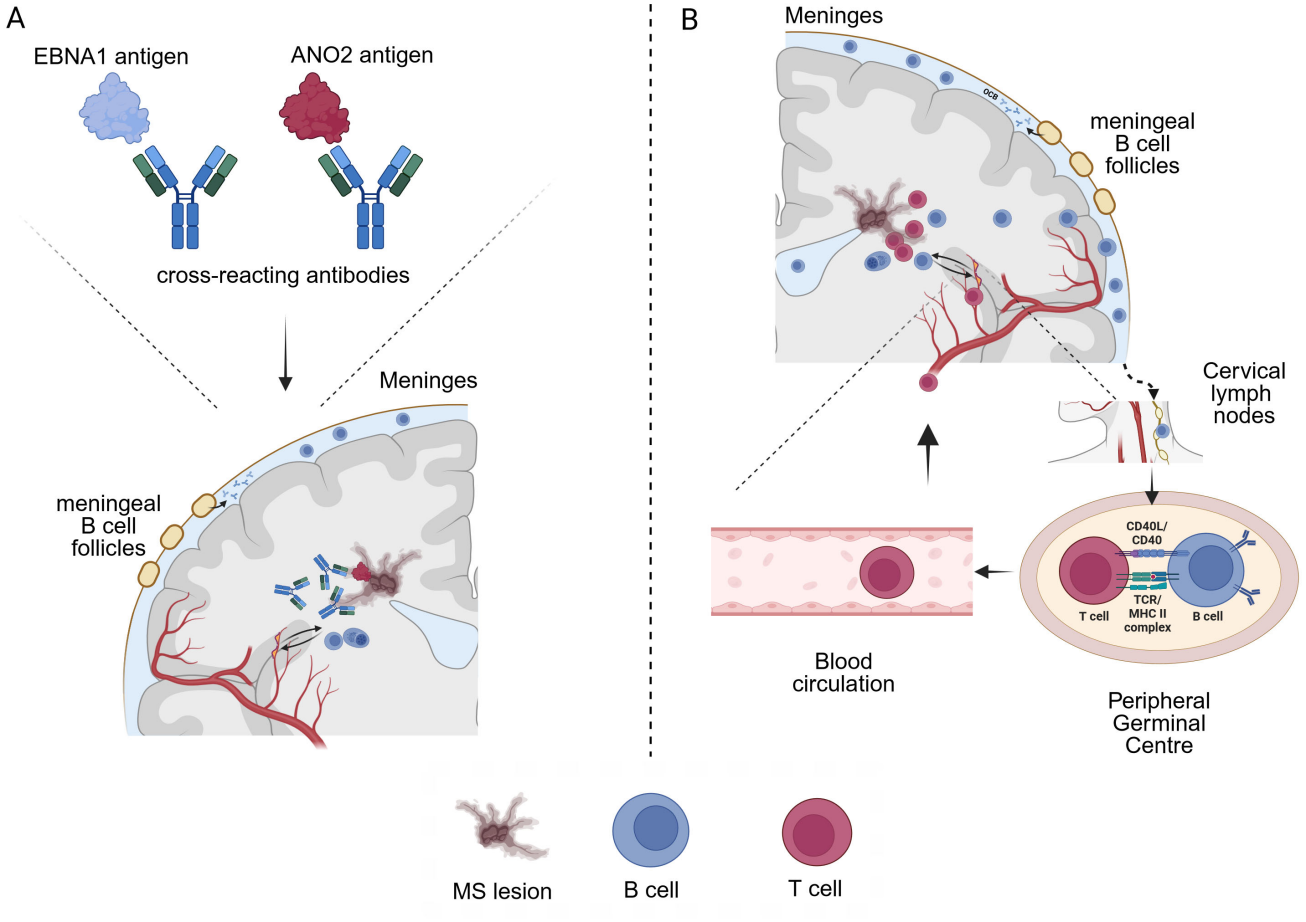
Two possible ways by which EBV infection could influence MS pathophysiology **(A)** Cross-reactivity (molecular mimicry) between EBNA1 and e.g. ANO2 peptides could lead to auto-reactive antibodies that cross the blood-brain-barrier and/or are produced locally by B cells and exert tissue damage (MS lesions). However, the exact pathophysiological role and ability of e.g. anti-ANO2 antibodies to activate CDC and ADCC mediated tissue damage has not been demonstrated yet. Of note, MS relapses do not seem to be associated with elevated ANO2 antibody titers. **(B)** A certain immune predisposition may influence long term EBV infected B cells that drive autoimmune responses through antigen presentation and thus promote CNS cross-reactivity of T cells. B cell trafficking in and out of the CNS has been demonstrated, with B-cell drainage through the cervical lymph nodes. B cells that have encountered CNS antigens during lesion formation could thus recirculate into the periphery and stimulate auto-reactive T cells. In this context, EBV infected B cells have been shown to efficiently process antigens for the presentation to CD4^+^ T cells via MHC class II (43).

For the different EBNA1 peptides and potentially cross-reacting peptides, we observed differential antibody reactions. All MS patient groups, and EBV-positive controls showed elevated anti EBNA antibodies against all EBNA1 proteins/peptides when compared to EBV-negative controls. Significantly elevated antibody titers of untreated RRMS patients were also observed against EBNA1 P72 (A), EBNA1 (AA386-405) and EBNA1 (AA425-444) when compared to EBV-positive controls. Interestingly, no significant difference was seen for EBNA1 P72 (S) which should be a similar protein to EBNA1 P72 (A). Our findings are, at least in part, consistent with existing literature indicating elevated antibody titers in MS patients relative to EBV-positive controls. However, it is notable that different test methods using different antigens yield disparate results, with anti-EBNA1 antibody titers not consistently demonstrating significantly elevated values in MS (50). In general, the C-terminal domain of EBNA1 was generally described as a carrier of for MS relevant epitopes so that the elevated antibody titers against EBNA1 (AA386-405) and EBNA1 (AA425-444) are in line with the literature (30, 31, 51). A significantly higher antibody titer to ANO2 was also found in untreated RRMS without relapse (37.8% positive) compared to EBV-positive controls (16.7% positive), which is consistent with previous findings that a significantly higher proportion of MS patients (approximately 14.5%) had antibodies compared to controls (17, 34, 40). Since the antibody response against EBNA1 (AA425-444) and ANO2 strongly correlated with each other, it might be speculated whether a specific response with further B cell maturation against ANO2 occurs or whether antibodies only demonstrate cross-reactivity between both targets (see **Figure 4**). In contrast, titers against EBNA1 (AA386-405) and GlialCAM were low (RRMS patients positive in 0%-2.6%), and we could not observe a MS specific antibody response (EBV-positive controls 2.8% positive for anti GlialCAM antibodies). These results partially contrast with other studies that found elevated anti GlialCAM antibodies in a subset of MS patients (36, 37). When looking at the antibody titer measurements of Lanz and colleagues, an ELISA test applying the described EBNA1 (AA386-405) and GlialCAM (AA370-389) peptides was used to analyse MS patients versus healthy controls within a patient collective of comparable or slightly lager sample size. Overall, statistical differences in antibody titers against EBNA1 (AA386-405) and GlialCAM (AA370-389) were observed at the group level, with only a subset of MS patients exhibited significantly elevated antibody titers (36). Although we observed a more consistent antibody response against CRYAB, especially in a subset of MS patients (RRMS patients positive in 22.2%-23.1%, versus 30.6% in EBV-positive controls), again no MS specific antibody response was detectable which again partially contrasts with previously published results that showed an increased CRYAB antibody response in MS (35). No treatment effects were observed on antibody titers.

Our results have certain implications regarding the pathophysiological role of EBV in MS. It is important to note that numerous studies have been performed on anti-EBV antibody responses in MS patients, with some inconsistent results most likely due to differences in patient populations, study designs, antigens and detection assays used. This is also reflected in this study where the same protein produced by two different manufacturers resulted in differing results, suggesting further investigation into these assay- and protein-specific differences in anti-EBV assays is needed. All of the patients with MS exhibited evidence of prior EBV infection, as indicated by the presence of antibodies against EBNA1 also showing a consistent anti-VCA IgG antibody response which has been consistently described in previous studies. It is important to note that no significant differences in antibody levels were observed between RRMS with or without relapses, SPMS or PPMS. The absence of elevated antibodies to immediate early or early antigens and a consistent IgM response suggests that a recent infection or pronounced EBV reactivation is not a major contributor during MS pathophysiology. Although antibody titers to EA were elevated in one previous study, no correlation with disease activity was found (15), and -in addition- several other studies have failed to demonstrate a consistent association between other EBV antibodies and MS (52). These results, in conjunction with the absence of disparities between RRMS patients with and without relapse in our study, indicate that EBV reactivation and the associated anti-EBV humoral immune response itself do not appear to trigger MS activity. As no clear differences in antibody signatures were observed in progressive disease phases compared to relapsing-remitting MS, an association between anti-EBV humoral immune response and disease progression cannot be established either. Nevertheless, MS specific elevated antibody responses were consistently observed against gh/gp42 fusion proteins and certain EBNA1 peptides, indicating a distinct humoral immune response to EBV in MS patients when compared to controls. One potential explanation for these differences may be linked to the prevalence of certain HLA alleles in MS (51). Indeed, interactions between HLA genotypes and reactivity to EBV-related epitopes suggest that the mechanism through which HLA genes influence MS risk may, at least partially, involve the immune control to EBV infection (53).

When examining cross-reactivity between EBNA1 epitopes, our results cannot rule out a certain role of an anti GlialCAM or CRYAB reactivity due to the limited number of patients but do not support an MS specific antibody response to those antigens. In contrast, our study provides further evidence that anti-ANO2 antibodies are specifically elevated in a subset of MS patients and correlate with anti EBNA1 (AA425-444) indicative for the described cross reactivity between both antigens. Further evidence for a possible role of ANO2 as a potential target results from previous studies that showed ANO2 expression near and inside of MS lesions as well as autoantibody reactivity against ANO2 (40). However, to the best of our knowledge, comprehensive pathological studies of anti-ANO2 antibodies, such as those conducted for anti-AQP4 antibodies in NMOSD (54), are lacking. Whether anti-ANO2 antibodies could indeed induce tissue damage in the CNS by CDC or ADCC is still unclear. Although our cross-sectional design might not be sufficient to measure minor changes in anti-EBV antibody levels on an individual patient basis over time, we did not observe significant effects on anti-EBNA1 and anti-ANO2 antibodies following treatments also including B cell depletion with OCR. This contrasts with effects of e.g. rituximab in NMOSD showing significant decreased anti AQP4 antibody titers after short- and long-term treatment (55).

Since our study does not provide information on functional aspects of anti-EBV antibodies, the exact role of elevated anti-ANO2 antibodies remains elusive but provides further evidence for a potential role in terms of molecular mimicry (**Figure 4A**). On the flipside, the differential antibody response against EBV in MS patients when compared to EBV-positive controls strongly suggests an altered immunological reaction in MS patients and possible effects during latent infection that could also influence MS pathophysiology. Other studies on EBV and MS have found not only elevated antibody titres against the most prominent target EBNA1, but also significantly elevated antibody titers against targets such as EBNA3, EBNA4, EBNA6 and LMP1 (30, 31). Although these were not investigated in the present study, we found significantly increased antibody titers against the docking/fusion proteins gh/gp42 and gh/gL/gp42 in MS patients, which complementary to the previous findings emphasise the role of other latent EBV proteins in MS. As a suggestion on the pathomechanism a possible imbalance between the host immune system and the virus was proposed (30). Going one step further, another study examining *ex vivo* gene expression in spontaneous lymphoblastoid cell lines from MS patients concluded that EBV-infected B cells expand during active disease thus promoting B and T cell inflammation (56). In this context, we hypothesize that a certain immune predisposition could influence long term EBV infected B cells that persist over time and drive autoimmune reactions by antigen presentation, promoting CNS cross reactivity of T cells. It has been demonstrated that EBV-infected B cells are capable of efficiently processing antigens for presentation to CD4^+^ T cells via MHC class II (57). Given that B cells have been shown to traffic in- and out of the CNS/CSF compartment (20, 58) EBV infected B cells that have encountered CNS antigens could thus feed autoimmune circuits (**Figure 4B**).

Several limitations of our studies have to be discussed. First, we performed a retrospective study and the patient groups exhibited differences in terms of patient characteristics, with age being a notable factor. Nevertheless, the observed differences between patient groups were consistent with the typical features of MS phases observed in clinical practice. Moreover, correlations between antibody titers and patient characteristics were scarce. No correlations were identified between patient characteristics and EBNA1 or corresponding cross-reactive peptides. Second, patient groups were sometimes small which limited the statistics in some cases. It also has to be noted that our patient collective was significantly smaller than some examined in the other cohorts (34, 35) so that our study might have been underpowered to reproduce those results and detect slight differences in antibody titers. Given the size of our major groups including RRMS and controls we still think that we can provide robust statistical analyses for antibody responses. A third limitation of the study is that it did not include longitudinal samples of treated MS patients. As a result, it was not possible to assess changes in individual antibody levels. Thus, our study approach may have failed to detect minor alterations in antibody titers. Forth, the antigens used in our test and our specific methodological approach using multiplex technology might have influenced our test results. However, results for some antigens were partially reproduced during test-establishment also using validated EBV ELISA tests. Fifth, the lack of access to samples with confirmed IgM-positive status introduces an element of uncertainty regarding the test results. This could potentially be attributed to either an insensitivity of the methods employed or a genuine seronegativity of the samples. Nevertheless, as the test assay has been subjected to exhaustive validation procedures, we are confident that it is also applicable to IgM subtypes.

In conclusion, our findings indicate a differential antibody response in MS patients regarding EBV antibody responses, particularly to EBNA1 peptides and gh/gp42 fusion proteins. These findings confirm an altered immunological reaction against EBV in MS patients. However, we did not observe signs for a pronounced EBV reactivation in MS or differential antibody signatures between different MS disease phases. Furthermore, elevated anti-ANO2 antibody titers were observed in MS patients when compared with EBV-positive controls, suggesting a potential role for EBNA1 and ANO2 antigens in terms of molecular mimicry. Nevertheless, the precise function of anti-ANO2 antibodies remains to be elucidated, and the specific role of EBV in MS pathogenesis still remains uncertain.

## Data Availability

The original contributions presented in the study are included in the article/**Supplementary Material**. Further inquiries can be directed to the corresponding author.
