# Supplementary material for: In-depth analysis of serum antibodies against Epstein-Barr virus lifecycle proteins, and EBNA1, ANO2, GlialCAM and CRYAB peptides in patients with multiple sclerosis
Source: Front Immunol. 2024 Dec 17;15:1487523. doi: 10.3389/fimmu.2024.1487523 (PMC11685087; doi:10.3389/fimmu.2024.1487523)
Supplement: Supplementary file 1 [file Table1.docx]

# **Supplementary Tables**

**Suppl. Table 1:** Additional description of characteristics within different patient groups in this study

|  | **Patient numbers** | **Age**  Mean in years  (Q1 – Q2 – Q3) | **Gender**  (f / m) | **Disease duration** Mean in months (Q1 – Q2 – Q3) | **Treatment duration** Mean in months (Q1 – Q2 – Q3) | **Number of previous DMTs**  Mean (Q1 – Q2 – Q3) | **Patients with relapse (n)** | **EDSS score** Mean  (Q1 – Q2 – Q3) |
| --- | --- | --- | --- | --- | --- | --- | --- | --- |
| EBV-negative control | 25 | 15.6 (11 – 14 – 22) | 10 / 15  (40% / 60%) | - | - | - | - | - |
| EBV-positive control | 36 | 31.6 (22 – 25 – 37.5) | 21 / 15  (42% / 58%) | - | - | - | - | - |
| Untreated RRMS during relapse | 39 | 29.0 (24 – 26 – 33) | 30 / 9  (77% / 23%) | 5.2 (0 – 1 – 2.5) | - | 0 (0 – 0 – 0) | 39 | 2 (1 – 1.5 – 2.5) |
| Untreated RRMS | 45 | 35.6 (26 – 30 – 47) | 34 / 11  (76% / 24%) | 54.3 (2 – 6.5 – 67.5) | - | 0.8 (0 – 0 – 1) | 0 | 2 (1 – 2 – 3) |
| Untreated SPMS | 17 | 49.7 (46 – 50 – 54) | 10 / 7  (59% / 41%) | 174.9 (97.5 – 158 – 227.5) | - | 1.6 (1 – 1 – 3) | 0 | 5 (4 – 4.5 – 6) |
| Untreated PPMS | 10 | 51.7 (49 – 52.5 – 53) | 5 / 5  (50% / 50%) | 47.2 (26 – 26 – 50) | - | 0.1 (0 – 0 – 0) | 0 | 4 (3 – 4 – 4) |
| RRMS glatiramer acetate | 11 | 29.3 (22.5 – 26 – 33.5) | 10 / 1  (91% / 9%) | 16.7 (9.5 – 12 – 15.5) | 9.3 (6 – 7 – 11) | 0.3 (0 – 0 – 0) | 6 | 2 (1 – 1.5 – 2) |
| RRMS teriflunomide | 13 | 42.0 (30 – 46 – 55) | 3 / 10  (23% / 77%) | 38.5 (10 – 16 – 32) | 10.0 (6 – 6 – 13) | 0.2 (0 – 0 – 0) | 5 | 2 (1.5 – 2 – 2) |
| RRMS dimethyl fumarate | 23 | 35.7 (26 – 36 – 41.5) | 16 / 7  (70% / 30%) | 27.1 (12 – 16.5 – 29.8) | 14.2 (6 – 11 –18) | 0.4 (0 – 0 – 1) | 7 | 2 (1 – 1.5 – 2.5) |
| RRMS cladribine | 19 | 33.4 (27.5 – 30 – 37.5) | 14 / 5  (74% / 26%) | 106.7 (42 – 64 – 138) | 21.2 (12 – 17 – 30) | 2.0 (1 – 1 – 3) | 3 | 3 (1.5 – 2.5 – 3) |
| RRMS ozanimod | 14 | 31.9 (27 – 31 – 37.8) | 8 / 6  (57% / 43%) | 12.4 (7 – 10 – 16) | 10.4 (6 – 6 – 16) | 0 (0 – 0 – 0) | 6 | 1.2 (1 – 1.5 – 1.5) |
| RRMS natalizumab | 10 | 34.6 (27 – 31 – 43) | 9 / 1  (90% / 10%) | 141.3 (48.3 – 146 – 181.3) | 43.5 (27.5 – 33 – 40.3) | 1.8 (1 – 2 – 2) | 3 | 4 (1.5 – 2 – 3) |
| RRMS ocrelizumab | 15 | 36.5 (27.5 – 32 – 45.5) | 10 / 5  (67% / 33%) | 94.3 (29 – 56 – 91) | 18.2 (7 – 12 – 23) | 1.0 (0 – 1 – 1) | 7 | 2 (2 – 2 – 2.5) |

**Suppl. Table 2:** EBV proteins / peptides used for antibody testing

| **Protein Group** | **EBV Antigens** | **Protein** | **Function and occurrence of antibodies** (for review, Debuysschere et al, 2023) |
| --- | --- | --- | --- |
| **Docking** | gp350/220  (12.5µg/mL) | Cambridge Bio  Cat. # 01-11-0035, Lot # 2022001 | Envelope protein gp350/220 leads to viral attachment to B cells via CD21 (receptor of complement C3d) and CD35. Neutralizing Abs against gp350, only appear during convalescent phase |
| **Fusion** | gH/gL/gp42  (12.5µg/mL) | Cambridge Bio  Cat # 01-11-0030, Lot # 2022001 | Fusion glycoproteins gH/gL lead to viral binding (integrins and HLAII) and promote membrane fusion. |
|  | gH/gp42  (12.5µg/mL) | Cambridge Bio  Cat # 01-11-0031, Lot # 2022001 |  |
| **IEA** (immediate early antigen) | BZLF1  (12.5µg/mL) | Biozol  Cat # ASB-OPCA02106, Lot # DA045029b1g0 | Initiation of lytic replication cycle is characterized by the initial expression of the BZLF1 gene and the production of Z Epstein-Barr replication activator protein (ZEBRA). anti-BZLF1 antibodies are considered to be markers of EBV reactivation |
| **EA** (early antigen) | EA P54  (31.3µg/mL) | Serion Immunologics  Cat # BA1363P54VS, Lot # A1363P54BI | Regulatory proteins synthesized to allow for production of viral DNA, structural proteins including viral capsid antigen and membrane proteins. EA IgG increases during the first 3-4 weeks (also during infectious mononucleosis) and is usually no longer detectable after 3-4 months. |
|  | EA P138  (12.5µg/mL) | Serion Immunologics  Cat # BA1363VSR6, Lot # A1363R6MH |  |
|  | EA P85 (restricted)  (12.5µg/mL) | Abcam  Cat # ab224863, Lot # 1045163-1 |  |
| **VCA** (viral capsid antigen) | VCA gp125  (93.8µg/mL) | Aviva Systems  Cat # Bio OPEF01335, Lot # L21670352 | Antibodies against the capsid antigen IgG (VCA IgG) typically appear at the time of the onset of the clinical symptoms of acute infection, and remain positive for life, whereas IgM antibodies (VCA IgM) usually appear at the same time as VCA IgG and disappear within a few weeks. |
|  | VCA P23  (12.5µg/mL) | Serion Immunologics  BA1361VSR21, A1361R21BG |  |
|  | VCA P18  (12.5µg/mL) | Serion Immunologics  BA1361VSR22, A1361R22MK |  |
| **EBNA1** (EBV nuclear antigens) late antigen | EBNA1_full protein  (31.3µg/mL) | Abcam  ab241243, 1059079-1 | EBNA1 plays a major role in the establishment of EBV latency and is expressed during all latent phases in immortalised cells, making it a prime target for the immune system. It is involved in viral DNA replication, expression of other latent genes, maintenance of EBV as episomal DNA, immune evasion of the virus and cell immortalisation. Anti-EBNA-1 IgG is usually undetectable during the first 3-4 weeks after the onset of clinical symptoms and is therefore indicative of past infection. Immunosuppressed patients are negative for EBNA-1 IgG or have only low levels. |
|  | EBNA1, p72 (A, Aviva) (12.5µg/mL) | Aviva Systems Bio  OPEF01346 |  |
|  | EBNA1, p72 (S, Serion) (12.5µg/mL) | Serion Immunologics  BA1362VS, A1362P72BN |  |
|  | EBNA1 AA386-405 | SQSSSSGSPPRRPPPGRRPF |  |
|  | EBNA1 AA393-412 | PPPGRRPFFHPVGEADYFEY |  |
|  | EBNA1 AA425-444 | GEPDVPPGAIEQGPADDPGE |  |
| **EBV cross reacting peptides** | GlialCAM AA370-389 | ATGRTHpSSPPRAPSSPGRSR* | Glial cell adhesion molecule, expressed on glial cells (* phosphorylated) |
|  | CRYAB AA2-21 | MDIAIHHPWIRRPFFPFHSP | Alpha crystallin B, expressed by oligodendrocytes |
|  | ANO2 AA134-153 | EPHAGGPGDIELGPLDALEE | Anoctamin 2, chloride-channel protein modulation for neural-excitability |

**Suppl. Table 3:** IgM positivity response rate for EBV lifecycle antigens

|  | **gp350/220** | **gH/gp42** | **gH/gL/gp42** | **BZLF1** | **EA P54** | **EA P138** | **EA P85** | **VCA P18** | **VCA P23** | **VCA gp125** | **EBNA1 full protein** |
| --- | --- | --- | --- | --- | --- | --- | --- | --- | --- | --- | --- |
| Negative Control (n = 25) | 0 / 0% | 0 / 0% | 0 / 0% | 0 / 0% | 0 / 0% | 0 / 0% | 0 / 0% | 0 / 0% | 0 / 0% | 0 / 0% | 0 / 0% |
| Positive Control (n = 36) | 3 / 8.3% | 6 /16.7% | 5 / 13.9% | 2 / 5.6% | 2 / 5.6% | 0 / 0% | 0 / 0% | 3 / 8.3% | 2 / 5.6% | 5 / 13.9% | 2 / 5.6% |
| RRMS UT RE (n = 39) | 1 / 2.6% | 3 / 7.7% | 2 / 5.1% | 0 / 0% | 0 / 0% | 0 / 0% | 0 / 0% | 0 / 0% | 0 / 0% | 2 / 5.1% | 0 / 0% |
| RRMS UT (n = 45) | 1 / 2.2% | 0 / 0% | 2 / 4.4% | 1 / 2.2% | 0 / 0% | 0 / 0% | 0 / 0% | 0 / 0% | 0 / 0% | 2 / 4.4% | 1 / 2.2% |
| SPMS UT (n = 17) | 0 / 0% | 0 / 0% | 0 / 0% | 0 / 0% | 0 / 0% | 0 / 0% | 0 / 0% | 0 / 0% | 0 / 0% | 1 / 5.9% | 1 / 5.9% |
| PPMS UT (n = 10) | 2 / 20.0% | 1 / 10.0% | 3 / 30.0% | 1 / 10.0% | 1 / 10.0% | 0 / 0% | 0 / 0% | 0 / 0% | 0 / 0% | 3 / 30.0% | 0 / 0% |
| RRMS GLAT (n = 11) | 1 / 9.1% | 1 / 9.1% | 0 / 0% | 0 / 0% | 0 / 0% | 0 / 0% | 0 / 0% | 0 / 0% | 0 / 0% | 0 / 0% | 0 / 0% |
| RRMS TER (n = 13) | 1 / 7.7% | 0 / 0% | 0 / 0% | 0 / 0% | 0 / 0% | 0 / 0% | 0 / 0% | 0 / 0% | 0 / 0% | 0 / 0% | 0 / 0% |
| RRMS DMF (n = 23) | 0 / 0% | 0 / 0% | 1 / 4.4% | 0 / 0% | 1 / 4.4% | 0 / 0% | 0 / 0% | 2 / 8.7% | 0 / 0% | 2 / 8.7% | 0 / 0% |
| RRMS CLAD (n = 20) | 0 / 0% | 1 / 5.0% | 2 / 10.0% | 0 / 0% | 0 / 0% | 0 / 0% | 0 / 0% | 0 / 0% | 0 / 0% | 1 / 5.0% | 0 / 0% |
| RRMS OZA (n = 14) | 0 / 0% | 0 / 0% | 0 / 0% | 3 / 21.4% | 0 / 0% | 0 / 0% | 0 / 0% | 1 / 7.1% | 0 / 0% | 0 / 0% | 0 / 0% |
| RRMS NAT (n = 10) | 0 / 0% | 0 / 0% | 0 / 0% | 0 / 0% | 0 / 0% | 0 / 0% | 0 / 0% | 0 / 0% | 0 / 0% | 0 / 0% | 0 / 0% |
| RRMS OCR (n = 15) | 0 / 0% | 1 / 6.7% | 1 / 6.7% | 0 / 0% | 0 / 0% | 0 / 0% | 0 / 0% | 0 | 0 / 0% | 1 / 6.7% | 0 / 0% |

Samples were classified as IgM positive when their MFI was greater than the mean of the negative control group plus 6x SD. Data is provided as both number of positive samples and corresponding percentage. Abbreviations: RRMS = relapsing remitting multiple sclerosis patients, GLAT = glatiramer acetate, TER = teriflunomide, DMF = dimethyl fumarate, CLAD = cladribine, OZA = ozanimod, NAT = natalizumab, and OCR = ocrelizumab.

**Suppl. Table 4:** IgM positivity response rate for EBNA1 proteins, peptides and related peptides

|  | **EBNA1 AA386-405** | **GlialCAM AA370-389** | **EBNA1 AA393-412** | **CRYAB AA2-21** | **EBNA1 AA425-444** | **ANO2 AA134-153** | **EBNA1 full protein** | **EBNA1, p72 (Serion)** | **EBNA1, p72 (Aviva)** |
| --- | --- | --- | --- | --- | --- | --- | --- | --- | --- |
| Negative Control (n = 25) | 0 / 0% | 0 / 0% | 0 / 0% | 0 / 0% | 0 / 0% | 0 / 0% | 0 / 0% | 0 / 0% | 0 / 0% |
| Positive Control (n = 36) | 0 / 0% | 1 / 2.7% | 2 / 5.5% | 1 / 2.7% | 1 / 2.7% | 1 / 2.7% | 2 / 5.5% | 3 / 8.3% | 4 / 11.1% |
| RRMS UT RE (n = 39) | 1 / 2.6% | 1 / 2.6% | 2 / 5.2% | 0 / 0% | 0 / 0% | 1 / 2.6% | 0 / 0% | 1 / 2.6% | 5 / 12.8% |
| RRMS UT (n = 45) | 0 / 0% | 2 / 4.4% | 1 / 2.2% | 0 / 0% | 0 / 0% | 1 / 2.2% | 1 / 2.2% | 0 / 0% | 3 / 6.7% |
| SPMS UT (n = 17) | 0 / 0% | 0 / 0% | 0 / 0% | 0 / 0% | 0 / 0% | 0 / 0% | 1 / 5.9% | 0 / 0% | 1 / 5.9% |
| PPMS UT (n = 10) | 0 / 0% | 0 / 0% | 1 / 10% | 0 / 0% | 1 / 10% | 0 / 0% | 0 / 0% | 1 / 10% | 2 / 20% |
| RRMS GLAT (n = 11) | 1 / 9.1% | 0 / 0% | 1 / 9.1% | 0 / 0% | 0 / 0% | 0 / 0% | 0 / 0% | 1 / 9.1% | 1 / 9.1% |
| RRMS TER (n = 13) | 0 / 0% | 0 / 0% | 0 / 0% | 0 / 0% | 0 / 0% | 1 / 7.7% | 0 / 0% | 0 / 0% | 0 / 0% |
| RRMS DMF (n = 23) | 0 / 0% | 1 / 4.3% | 1 / 4.3% | 1 / 4.3% | 1 / 4.3% | 1 / 4.3% | 0 / 0% | 1 / 4.3% | 2 / 8.7% |
| RRMS CLAD (n = 20) | 0 / 0% | 0 / 0% | 1 /5.0% | 0 / 0% | 0 / 0% | 0 / 0% | 0 / 0% | 0 / 0% | 1 / 5.0% |
| RRMS OZA (n = 14) | 0 / 0% | 0 / 0% | 0 / 0% | 0 / 0% | 0 / 0% | 0 / 0% | 0 / 0% | 0 / 0% | 0 / 0% |
| RRMS NAT (n = 10) | 0 / 0% | 0 / 0% | 1 / 10.0% | 0 / 0% | 0 / 0% | 0 / 0% | 0 / 0% | 1 /10.0% | 0 / 0% |
| RRMS OCR (n = 15) | 0 / 0% | 0 / 0% | 0 / 0% | 0 / 0% | 0 / 0% | 0 / 0% | 0 / 0% | 0 / 0% | 0 / 0% |

Samples were classified as IgM positive when their MFI was greater than the mean of the negative control group plus 6x SD. Data is provided as both number of positive samples and corresponding percentage. Abbreviations: RRMS = relapsing remitting multiple sclerosis patients, GLAT = glatiramer acetate, TER = teriflunomide, DMF = dimethyl fumarate, CLAD = cladribine, OZA = ozanimod, NAT = natalizumab, and OCR = ocrelizumab.
