# Supplementary figures and images for: In-depth analysis of serum antibodies against Epstein-Barr virus lifecycle proteins, and EBNA1, ANO2, GlialCAM and CRYAB peptides in patients with multiple sclerosis
Source: Front Immunol. 2024 Dec 17;15:1487523. doi: 10.3389/fimmu.2024.1487523 (PMC11685087; doi:10.3389/fimmu.2024.1487523)

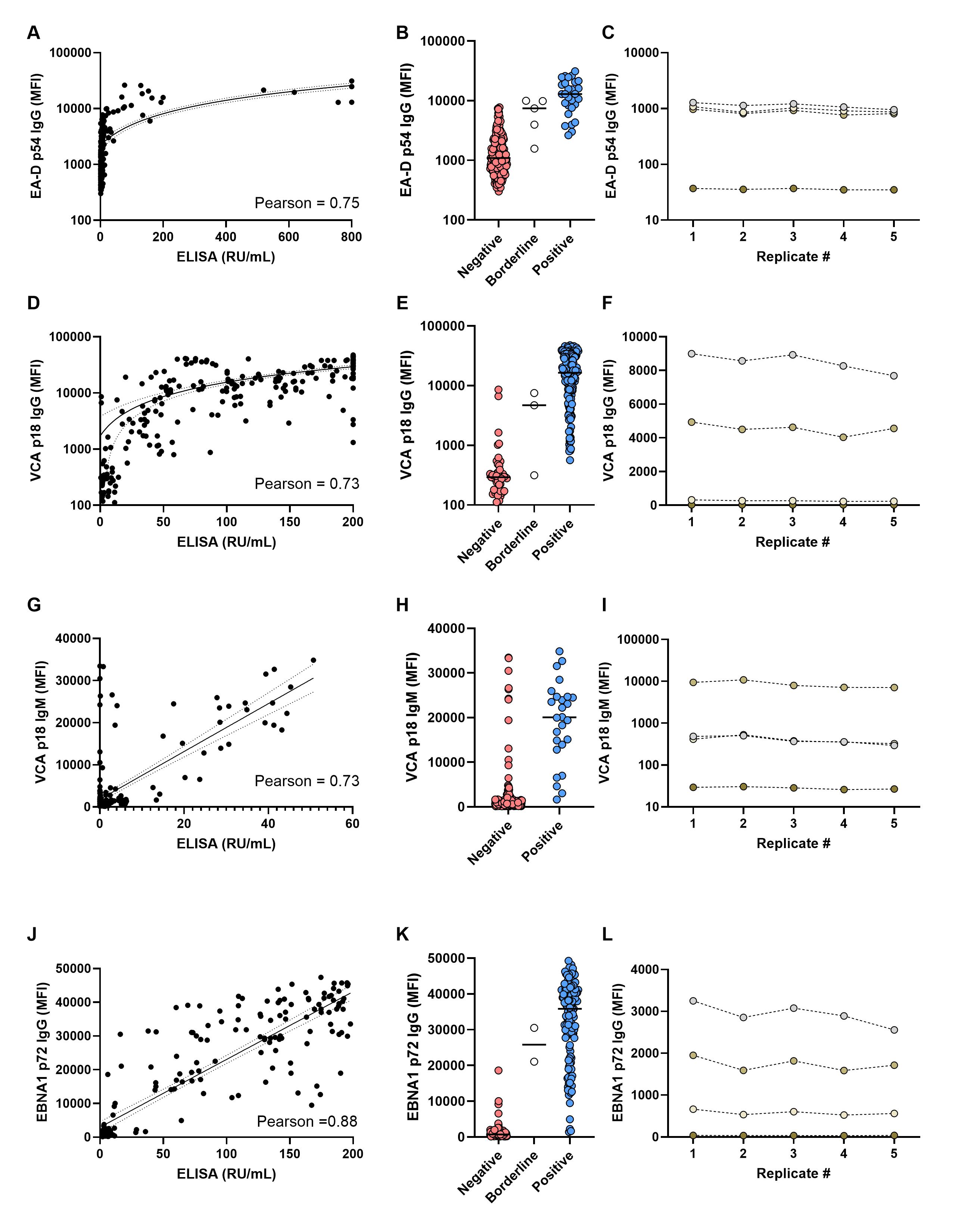

Supplement: Supplementary file 2 [file Image1.jpeg]

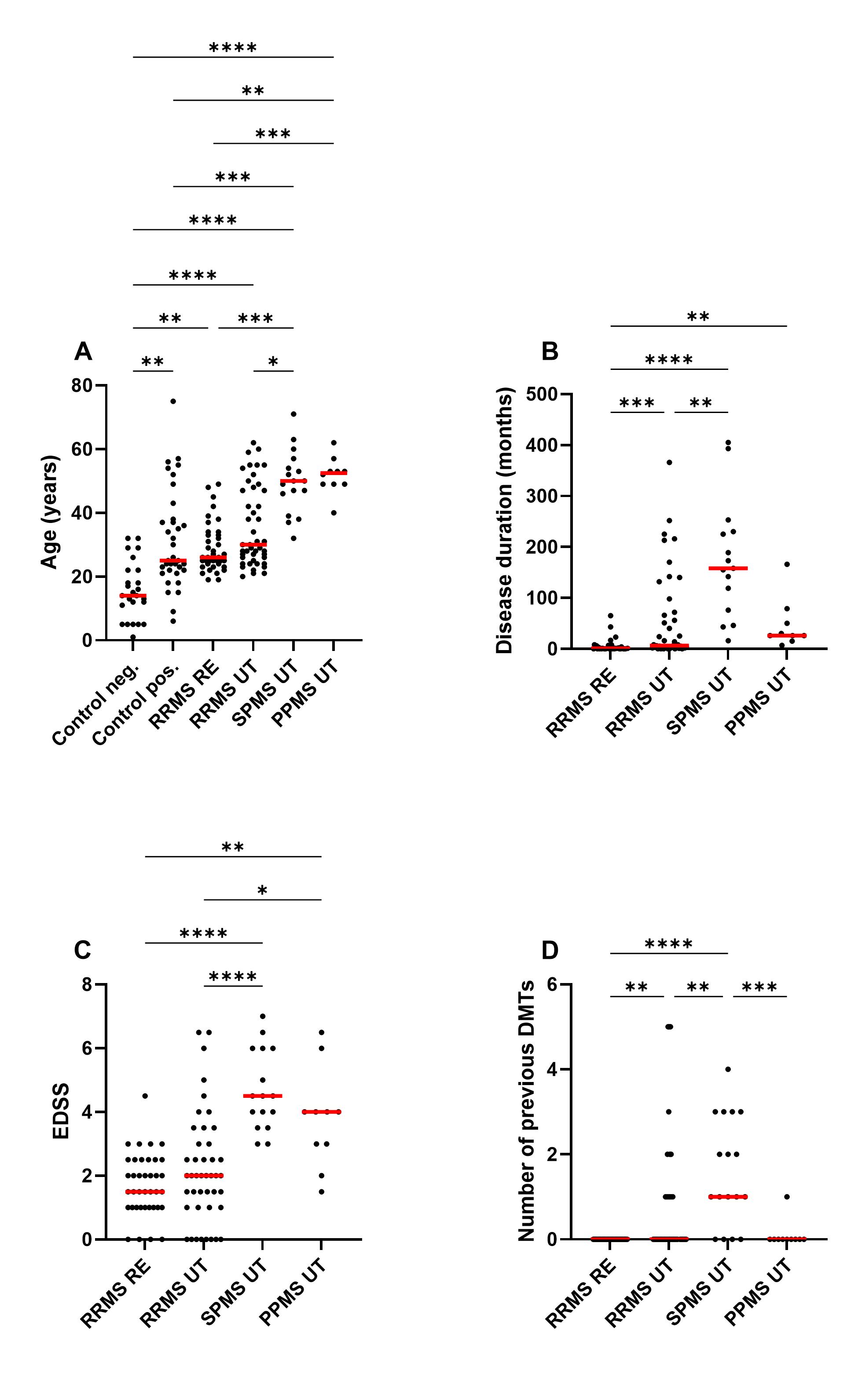

Supplement: Supplementary file 3 [file Image2.jpeg]

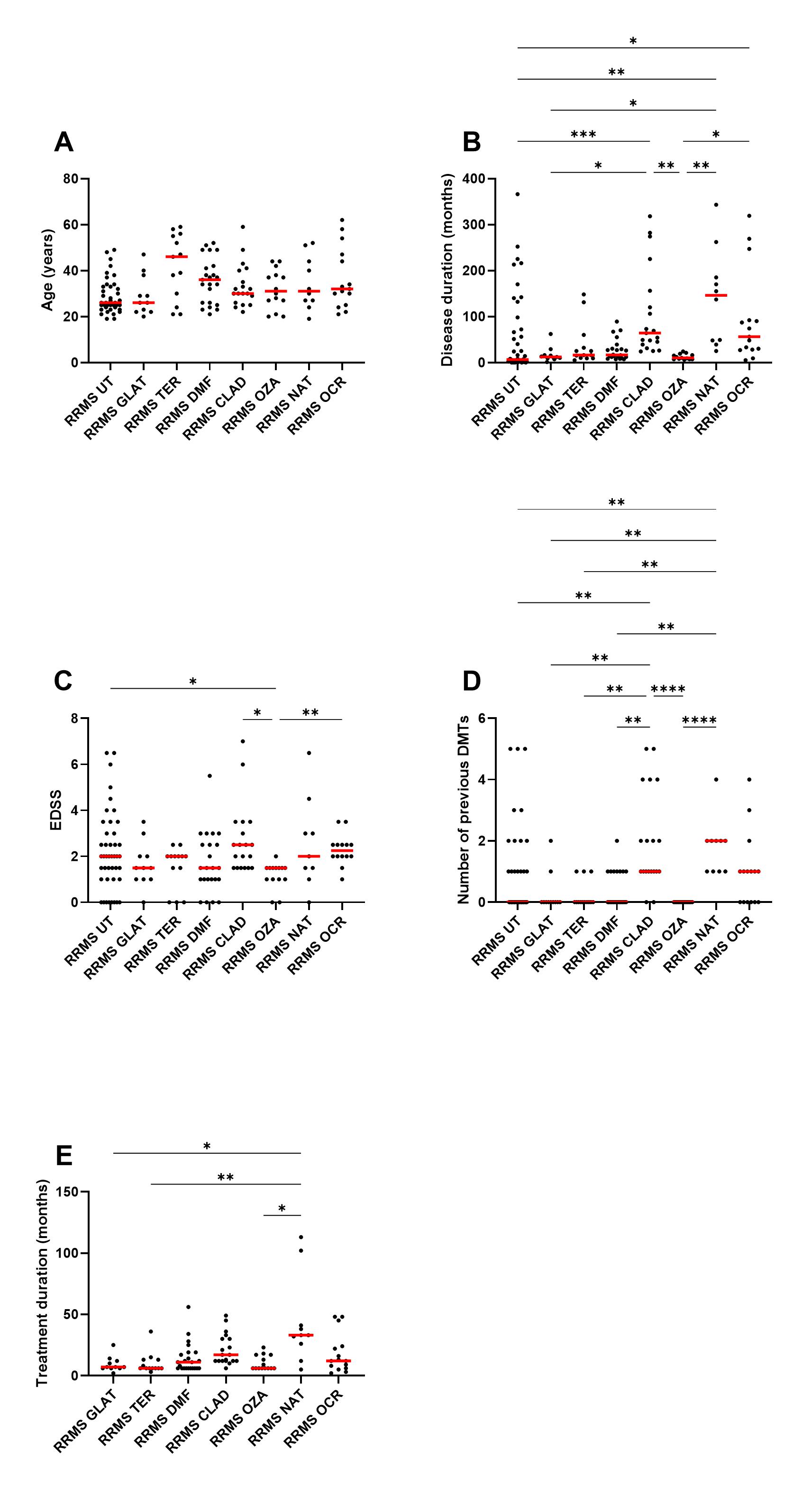

Supplement: Supplementary file 4 [file Image3.jpeg]

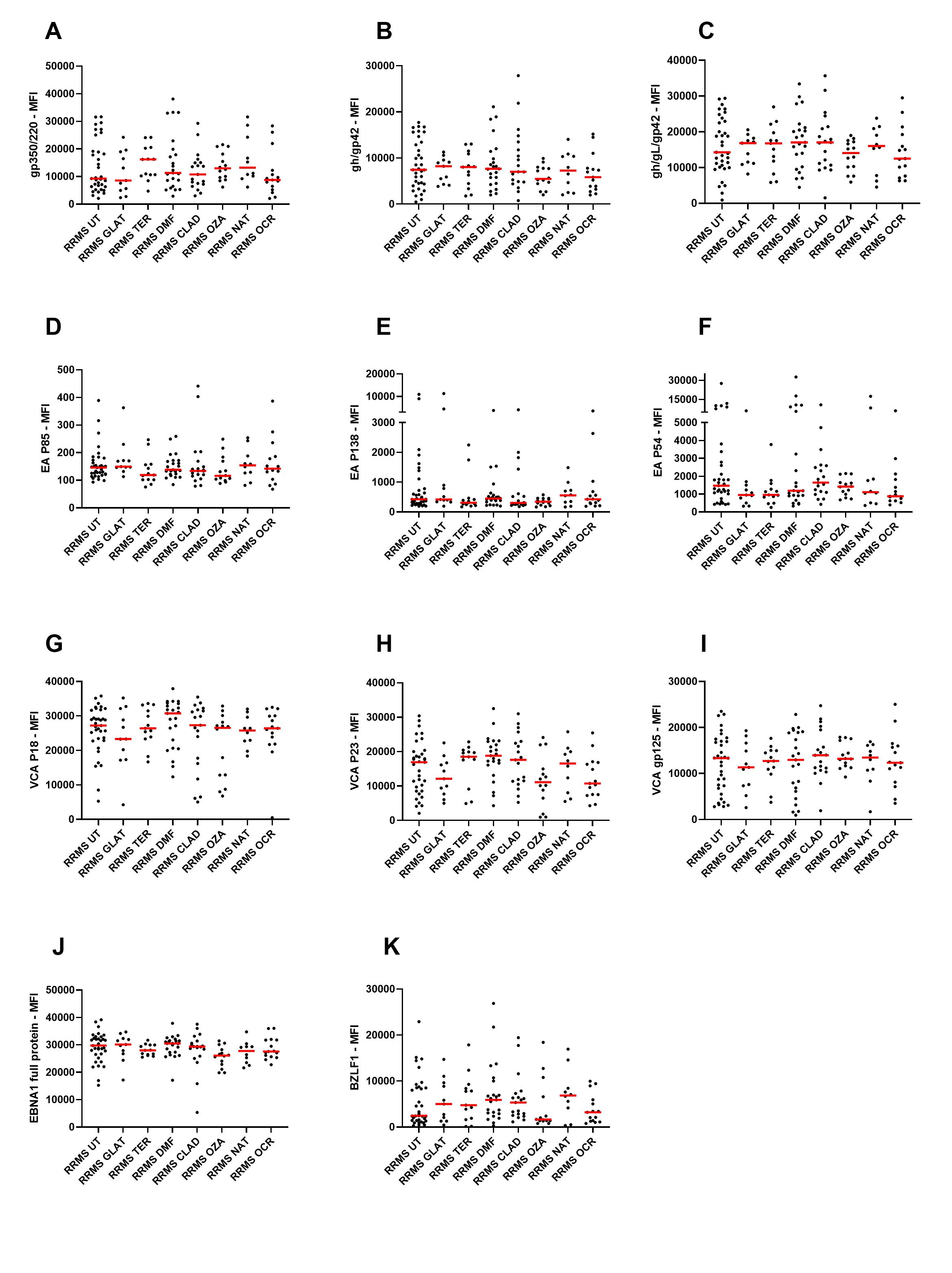

Supplement: Supplementary file 5 [file Image4.jpeg]

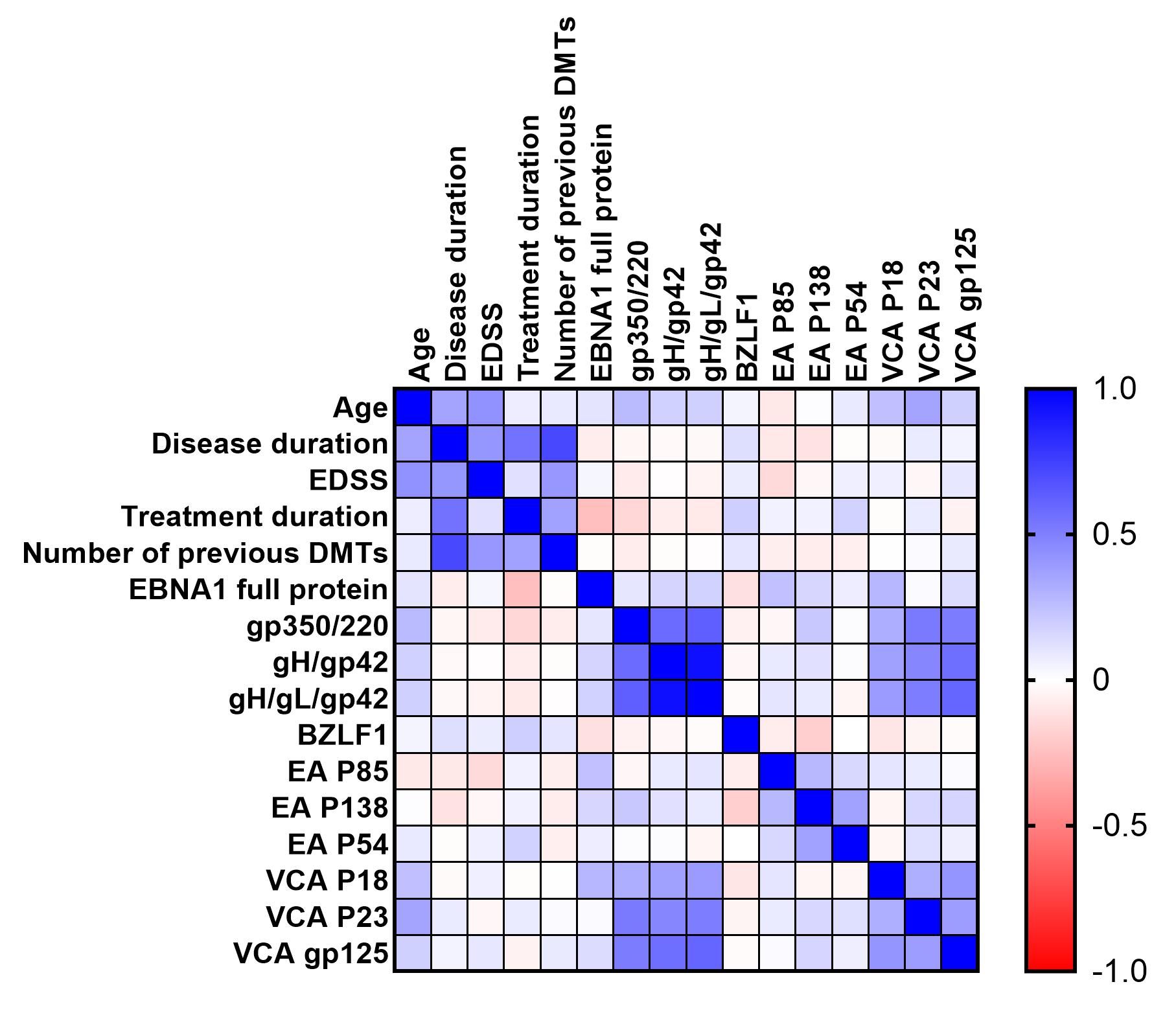

Supplement: Supplementary file 6 [file Image5.jpeg]

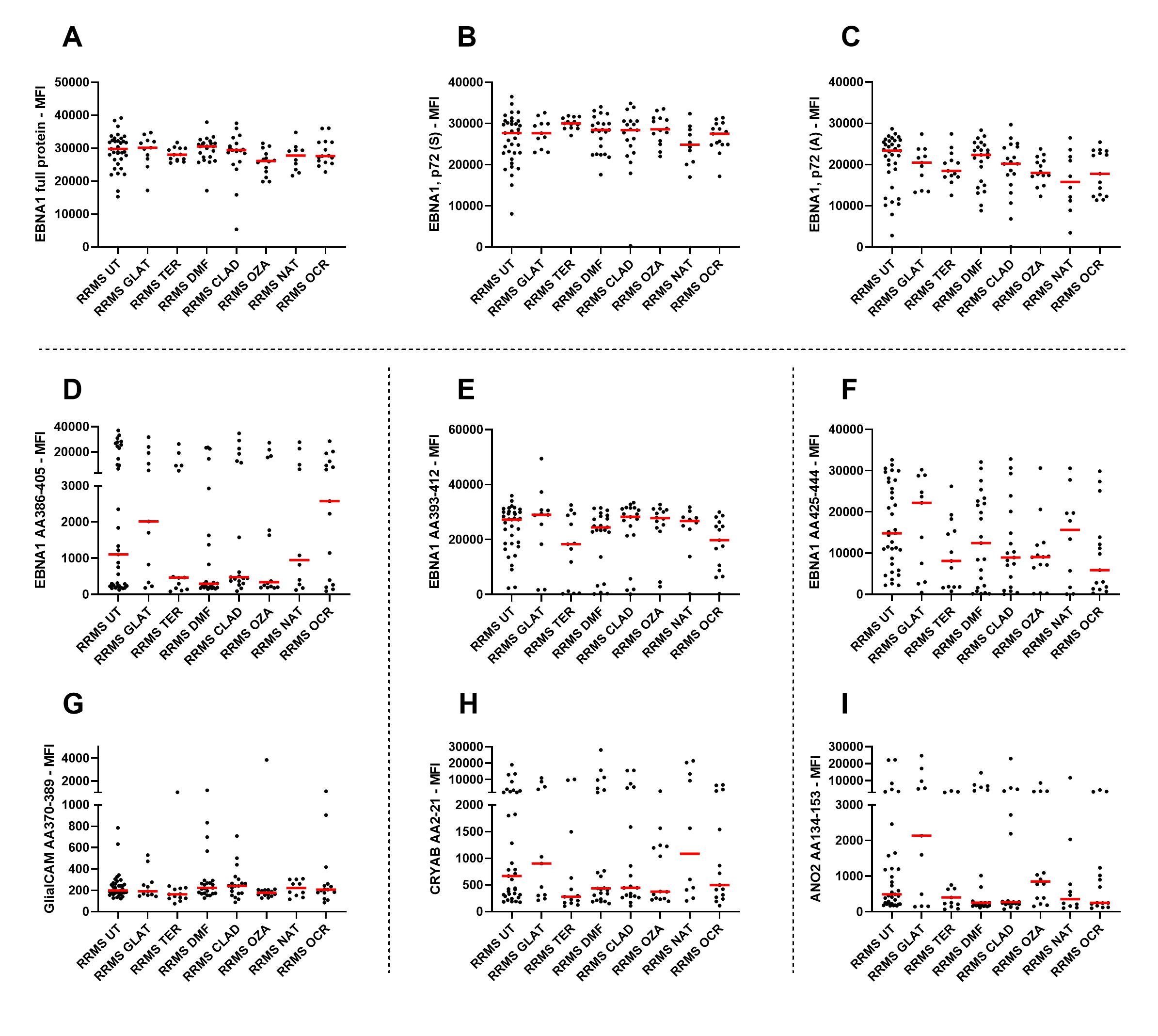

Supplement: Supplementary file 7 [file Image6.jpeg]
